# Infectious Scleritis Masquerading as Isolated Orbital Cellulitis: A Case Report

**DOI:** 10.7759/cureus.85714

**Published:** 2025-06-10

**Authors:** Yazeed J AlHaqbani, Almoayad M Makrami, Fatimah N Alzaher, Majed F Alsubaie, Abdulaziz S Khoshaim

**Affiliations:** 1 Ophthalmology, Dhahran Eye Specialist Hospital, Dhahran, SAU

**Keywords:** cellulitis, infectious, masquerading, orbital, scleritis

## Abstract

A woman in her fifties was presented to the emergency room with eye pain, swelling, redness, and reduced vision. The examination revealed periorbital swelling, limited extraocular motility, tenderness, and conjunctival chemosis, with thinning observed nasally at the site of prior pterygium repair. The anterior chamber was quiet, and B-scan (ultrasonography) revealed retino-choroidal thickening. Computed tomography (CT) scan results indicated septal cellulitis with clear sinuses. Laboratory workups were negative. The patient was diagnosed with orbital cellulitis and was admitted and treated accordingly. However, the patient developed recurrent attacks with hypopyon, which prompted another course of management. The patient was presented again, displaying an aggressive clinical picture, and there was a focal calcified plaque over the nasal area, suggesting necrotizing scleritis. B-scan shows T-sign and conjunctival recession, scleral scraping, debridement, and subconjunctival antibiotic injection were performed. A positive culture for Pseudomonas aeruginosa was identified. An agreement on the management of this ocular condition lacks a clear guideline. However, most infectious scleritis cases mandate a combined approach involving medical treatment and surgical debridement.

## Introduction

Scleritis refers to inflammation impacting the sclera, the outer layer of the globe [1]. The symptoms of scleritis may range from localized or diffuse redness and pain to severe visual impairment [1]. Although autoimmune activities are the most common etiology behind developing scleritis, approximately 5% to 10% of cases arise from infectious sources [1,2].

The clinical presentation of both infectious and autoimmune scleritis can share similarities, potentially leading to the mismanagement of infectious cases as autoimmune, thereby potentially worsening outcomes [1]. Most cases of infectious scleritis had a history of predisposing factors, particularly previous ocular surgery, especially pterygium excision. Other associated factors include prior accidental eye injury, exposure to radiation, and the use of mitomycin.

Despite various organisms being identified as causative agents in infectious scleritis, Pseudomonas aeruginosa remains the most commonly identifiable organism [1]. Managing most infectious scleritis cases mandates a combined approach involving medical treatment and surgical debridement, as evidenced by multiple studies demonstrating favorable outcomes through this integrated strategy [2]. Since the combined approach results in faster recovery than medical management alone, currently, an agreement on the management of this severe ocular condition is lacking a clear guideline [3]. However, initiating empirical broad-spectrum topical and systemic antibiotic therapy as a preemptive measure while awaiting laboratory results, with adjustments as warranted, is recommended [4]. The prognosis of infectious scleritis is determined by multiple factors, where poor initial vision, necrotizing type, and fungal cause usually bring worse outcomes [5].

Here, we present a case of severe infectious scleritis caused by Pseudomonas aeruginosa misdiagnosed as isolated orbital cellulitis.

## Case presentation

A woman in her fifties who is known to have type 2 diabetes, hypertension, and dyslipidemia. The patient was presented at the emergency department with left eye pain, swelling, and redness in the upper and lower eyelids, escalating over the past five days. She mentioned associated headaches, while other systemic reviews were normal. She had a history of pterygium excision for a decade, and recently, she was taking oral steroids and antihistamines for allergies, shrimp and egg allergies, which her dermatologist prescribed. Upon examination, the patient demonstrated stable vital signs. Her right eye exhibited normal findings, while the left eye had reduced vision 20/100 on the E chart, normal intraocular pressure, and a reactive pupil. The ophthalmological assessment revealed limited extraocular motility, severe left periorbital swelling, redness, and tenderness. A slit lamp examination of the left eye indicated diffuse conjunctival chemosis, with thinning observed nasally at the site of prior pterygium repair. The remaining part of her anterior segment examination was normal, with challenges in fundus visualization due to patient cooperation.

Investigations included a B-scan revealing a clear vitreous with retino-choroidal thickening in the left eye. The computed tomography (CT) scan results indicated pre-septal and septal cellulitis in the left eye with clear sinuses. The differential diagnosis considered orbital cellulitis versus endogenous endophthalmitis.

Despite negative results of septic and uveitis workup, the patient's symptoms responded to intravenous broad-spectrum antibiotics treatment, which included 500 mg of Metronidazole IV four times daily, as well as 400 mg IV Ciprofloxacin three times daily, with improvement in periorbital swelling leading to discharge on the third day with oral and topical antibiotics. The topical antibiotics were Cefazolin 25 mg/mL and Ceftazidime 25 mg/mL, four times daily each. The oral antibiotic regimen included Amoxicillin-Clavulanic Acid 1 g twice daily for 10 days, Metronidazole 500 mg three times daily, and Doxycycline 100 mg four times daily. However, six days later, the patient returned to the emergency room with worsened symptoms, including anterior chamber reaction, hypopyon, fibrinous reaction, and posterior synechiae formation.

**Figure 1:**
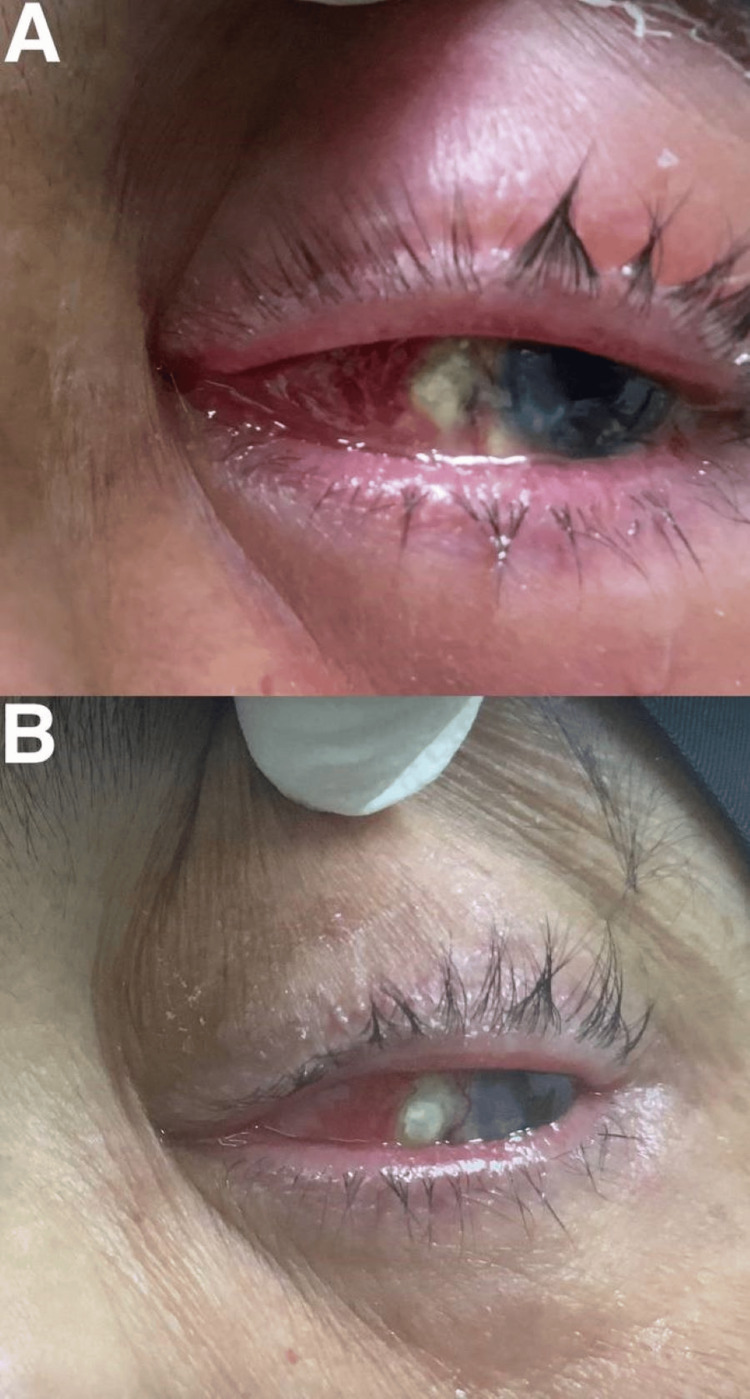
External photo: (A) on third emergency visit; (B) after proper diagnosis and management.

**Figure 2:**
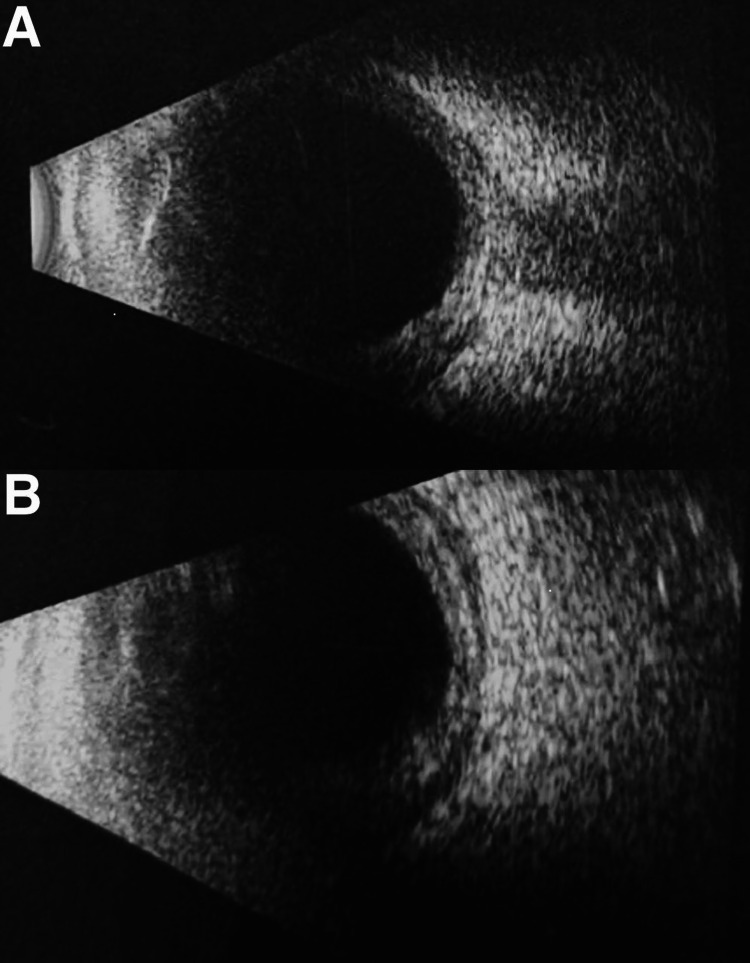
B-scan ultrasonography: (A) classic T-sign; (B) thickened sclera with fluid collection in the sub-Tenon space.

**Figure 3:**
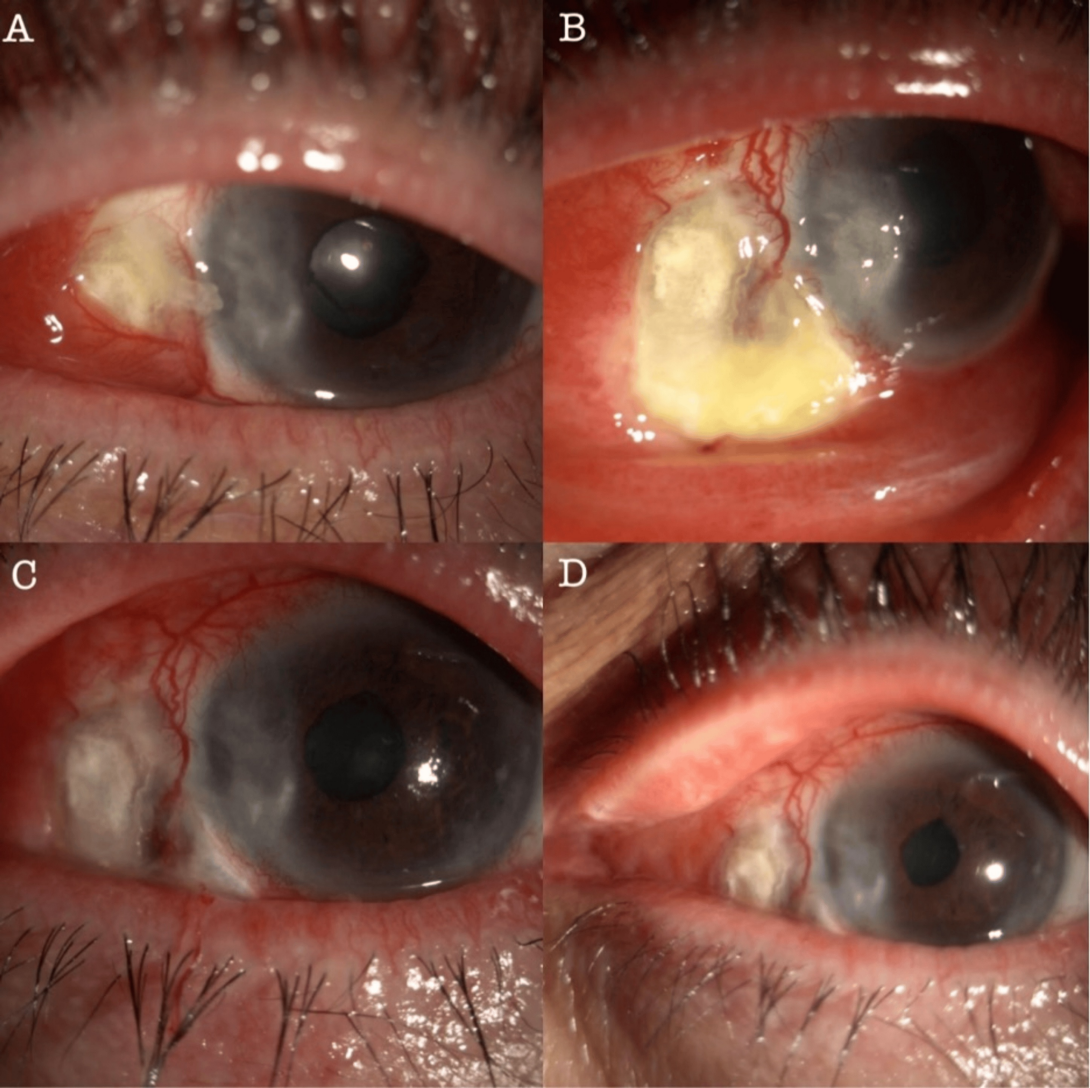
Slit-lamp photographs: (A) at presentation; (B) after conjunctival recession and scleral debridement; (C) at one-week follow-up; (D) at one-month follow-up.

The patient was then extensively investigated for systemic and local inflammatory causes, but the results returned negative. The patient was readmitted to receive another course of intravenous and topical antibiotics, which included 500 mg of Metronidazole IV four times daily for 10 days, as well as 400 mg IV Ciprofloxacin three times daily along with oral and topical steroids; the patient showed signs of improvement with last visual acuity reaching 20/30 on E-chart. One month later, the patient was presented to the emergency department again after completing her medications, displaying an aggressive clinical picture. The vision deteriorated to light perception. There was severe left eyelid swelling, conjunctival injection, and a focal calcified plaque over the thinned nasal scleral area, suggesting necrotizing scleritis (Figure 1). The B-scan indicated a typical T-sign for posterior scleritis (Figure 2), and the CT scan revealed clear sinuses with soft tissue swelling. A diagnosis of orbital cellulitis secondary to infectious scleritis was established. The patient underwent urgent conjunctival recession, scleral scraping, debridement, and subconjunctival antibiotic injection under general anesthesia (Figure 3). A positive culture for Pseudomonas aeruginosa sensitive to ciprofloxacin and ceftazidime prompted medication adjustments.

The patient exhibited significant improvement and received subconjunctival ceftazidime upon discharge. After one month, the patient's scleritis had resolved, and an amniotic membrane transplantation was planned.

## Discussion

Scleritis is a painful and potentially sight-threatening condition characterized by inflammation of the sclera and episclera, possibly involving adjacent structures like the cornea and uveal tissue. Immune-mediated disorders are often linked to scleritis, and around 40% of patients have an associated underlying systemic disease [4].

Infectious scleritis is a rare source of scleral inflammation, accounting for a 5% to 10% prevalence. Its rarity and similarity of its symptoms to non-infectious forms pose a challenge, as initiating corticosteroid treatment may exacerbate tissue damage and delay the intervention for infectious scleritis, contributing to its poor prognosis [6].

Clinical indicators differentiating infectious and immune-related scleritis include systemic disease and abnormal serological tests, suggesting immune-mediated scleritis. On the other hand, a history of trauma or surgery is associated with infectious scleritis. Additional signs for immune-mediated scleritis involve a bluish hue (localized or diffuse), bilaterality, past episodes noting a bluish hue, areas of thinning, and improvement with corticosteroids. Conversely, infectious scleritis may manifest with nodules (pus points, multifocal, ulcerated conjunctiva, underlying yellowish hue), mucopurulent discharge (accompanied by other factors like keratitis, keratic precipitates, and hypopyon), or a lack of response to corticosteroids followed by worsening [7].

Infectious scleritis can result from bacterial, viral, fungal, or parasitic agents, typically affecting compromised tissue due to disease or trauma. A comprehensive systemic review indicates that the predominant infectious culprits are varicella-zoster and Treponemal infections. A notable predisposing factor for infectious scleritis is a history of pterygium surgery involving mitomycin C or beta irradiation, with Pseudomonas aeruginosa as the most prevalent associated pathogen [8].

In a retrospective review conducted at tertiary eye centers in Saudi Arabia, 52 patients were involved in the study. Most scleritis cases involving 31 patients (59.6%) were idiopathic. Systemic associations were found in 23.1% of cases, with six patients having rheumatoid arthritis, four with Wegener's granulomatosis, and two patients with Vogt-Koyanagi-Harada (VKH) disease. Infectious scleritis was noted in 11.5% of cases, including 3 with bacterial scleritis, 2 with tuberculosis-related scleritis, and 1 with scleritis resulting from herpes simplex infection. Among all cases reviewed, four patients (7.6%) had a history of ocular surgery before the scleritis diagnosis. Remarkably, 3 out of 4 patients who had undergone previous pterygium excision were all of those three patients diagnosed with necrotizing scleritis caused by a bacterial infection [9].

Infectious scleritis can result in severe ocular complications such as glaucoma, cataracts, vitreous opacity, serous retinal or choroidal detachment, and even necessitate enucleation. A retrospective study involving 16 patients who developed infectious scleritis after pterygium excision revealed two groups: one receiving only medical treatment and the other undergoing surgical debridement in addition to medical treatment. Among the medical treatment group, two out of nine patients maintained vision ≥20/200, while five out of seven achieved vision ≥20/200 in the combined treatment group. The combined treatment approach effectively reduced the management duration and improved visual results [10].

## Conclusions

Infectious scleritis may present with symptoms and signs similar to orbital cellulitis. Considering infectious scleritis as a differential diagnosis, especially in patients with a history of pterygium excision and no signs of sinusitis or subperiosteal abscess, can help prevent delays in management and aid in disease resolution. The lack of a standardized treatment guideline for infectious scleritis makes management challenging, with a high recurrence rate. This study demonstrates that a combined medical and surgical approach can be practical.
